# Confidential Conversations in Palliative Care: An Ethnographic Exploration of Trust and Interpersonal Relationship Between Nurse and Patient

**DOI:** 10.1111/jocn.70119

**Published:** 2025-09-30

**Authors:** Tove Stenman, Bodil Holmberg, Ylva Rönngren, Ulla Näppä, Christina Melin Johansson

**Affiliations:** ^1^ Department of Health Sciences, Nursing Science Mid Sweden University Östersund Sweden; ^2^ Department of Nursing Science Sophiahemmet University Stockholm Sweden; ^3^ Department of Health Sciences, Nursing Science Mid Sweden University Sundsvall Sweden

**Keywords:** confidential conversations, end‐of‐life care, ethnography, interpersonal relations, nurse–patient relations, nursing, palliative care, qualitative research

## Abstract

**Aim:**

To explore aspects of interpersonal relationships in palliative care nursing, focusing on confidential conversations between patients and registered nurses (RN).

**Design:**

A qualitative study employing focused ethnography.

**Methods:**

Data were collected through unstructured participant observations, field notes and interviews with patients and RN in specialist palliative care. Data were analysed using reflective thematic analysis.

**Findings:**

Confidential conversations in palliative care are founded on trust that is fragile and develops dynamically through consistent interactions. Small talk, presence and silence are essential for initiating and maintaining trust and the interpersonal relationship. The environment, patient condition and RN emotional presence and competence shape these conversations. As the relationship evolves, conversations adapt to the patient's changing needs. Missed signals or interruptions can disrupt flow, but the potential for repair remains, allowing for restoration and strengthening of trust and connection.

**Conclusion:**

Confidential conversations in palliative care are grounded in fragile, dynamic trust, necessitating ongoing presence, sensitivity and adaptability from RN. To support these interactions, healthcare environments must prioritise privacy, relational continuity and communication training. Future research should investigate how organisational structures and clinical settings influence confidential conversations.

**Implications for the Profession and/or Patient Care:**

Healthcare environments should facilitate confidential conversations by ensuring relational continuity and minimising distractions. Communication training that emphasises presence and management of silence can strengthen nurse–patient relationships, enhancing patient care and emotional support.

**Impact:**

This study explores key aspects of confidential conversations in palliative care, emphasising trust and emotional sensitivity. It addresses a research gap in palliative care using rare observational methods to deepen understanding of nursing relational aspects. The findings offer practical guidance for enhancing communication and relational skills, informing training and policy development and ultimately, improving emotional support and care.

**Reporting Method:**

Findings are reported in accordance with the Consolidated Criteria for Reporting Qualitative Research guidelines.

**Patient or Public Contribution:**

This study did not involve patient or public participation in its design, conduct or reporting.

**Abbreviations**
RN: Registered Nurse
RTA: Reflexive Thematic Analysis
WHO: World Health Organization

**Summary**

- What does this paper contribute to the wider global clinical community?
  ○ Highlights the importance of relational skills—such as small talk, silence and conversational attunement—in building trust and emotional safety in palliative care
  ○ Demonstrates how strengthening these skills enhances person‐centred care and emotional well‐being at the end of life.
  ○ Contributes to a broader understanding of emotional communication in nursing, with relevance across various care contexts.

## 1 Introduction

For dying patients, the need to be heard, seen and understood is essential. Between clinical routines and everyday small talk, moments of trust unfold—unexpected and vital in palliative care. In the quiet spaces of care, ordinary conversations between patients and registered nurses (RNs) can evolve into confidential conversations where fears, hopes and reflections are shared (Macdonald 2016; Stenman et al. 2023; Tarbi et al. 2021).

In this study, confidential conversations refer to spontaneous, trust‐based exchanges that go beyond everyday small talk. These conversations offer patients space to handle unresolved concerns and reflect on their impending death (Stenman et al. 2023). They may arise spontaneously, sparked by a patient's readiness or a simple exchange during care (Macdonald 2016; Tarbi et al. 2021). Confidential conversations offer relief and support (Cribb et al. 2022; Engel et al. 2023; Stenman et al. 2024), while also challenging RNs emotionally, requiring sensitivity, presence and careful navigation of professional boundaries (Höglander et al. 2023).

Palliative care aims to relieve suffering and improve quality of life for people with life‐limiting conditions (World Health Organization [WHO] 2020). As the global need for such care increases (WHO 2020), supporting patients emotionally and existentially remains a challenge across care settings (Ohinata et al. 2022). Confidential conversations are one form of support, but they can be unpredictable and demanding for RNs. Greater understanding of how these interactions develop may inform clinical training and policy across different cultural and organisational contexts.

While communication in palliative care has been explored, less is known about how everyday interactions evolve into trustful, confidential conversations. These moments, shaped by the RN's presence and the patient's openness, can contribute to support within care relationships. Further insight into these conversations may help improve the support at the end of life.

## 2 Background

Palliative care supports individuals with life‐threatening or life‐limiting conditions by alleviating suffering and enhancing quality of life. It addresses physical, psychological, social and existential needs through person‐centred care (Payne et al. 2022). Globally, an estimated 56.8 million people require palliative care, including 25.7 million in their final year of life—a number projected to rise due to ageing populations and chronic illnesses (WHO 2020). Care is delivered across various settings, including hospitals, home care, hospices and consultation services, involving both generalist and specialist teams. Regardless of context, the focus is on supporting patients and families when a cure is no longer possible (Payne et al. 2022). This complex care environment creates both opportunities and challenges (Ohinata et al. 2022).

The inevitability of death is a universal aspect of life and confronting it can be challenging (Croker et al. 2022). Patients with palliative care needs face physical, emotional, social and existential difficulties. As life nears its end, the need for meaning, support and understanding intensifies (Hussain 2020). Addressing these complex needs requires a person‐centred approach, where patients' experiences, wishes and values guide care (Öhlén and Friberg 2023) as supportive care aims to enhance quality of life at the end of life (Oberle and Davies 1993).

The interpersonal relationship between RNs and patients, grounded in trust, compassion and active listening, is highlighted in a review of RN–patient communication (Höglander et al. 2023). This relationship supports person‐centred care, as shown in both a meta‐synthesis (Croker et al. 2022) and a review of palliative nursing values (Moran et al. 2021), where both parties influence each other in ways that can be supportive and challenging (Oberle and Davies 1993; Smith et al. 2024).

A grounded theory study demonstrated how the RN's role extends beyond clinical expertise, integrating compassion to deliver person‐centred care in palliative contexts (Lagerin et al. 2025). In a concept analysis of the caring encounter, Holopainen et al. (2019) described how conversations deepen interpersonal relationships and create space for reflection, aligning with the emphasis on open communication in supportive care (Oberle and Davies 1993). From patients' perspectives, a review showed that patients with palliative care needs seek affirmation in their suffering, which confidential conversations can provide (Engel et al. 2023). These conversations allow patients to express fears and concerns (Tarbi et al. 2021), offering relief and a sense of meaning (Cribb et al. 2022; Oberle and Davies 1993).

Conversations require RNs to adapt with compassion and relational sensitivity (Höglander et al. 2023). Presence is essential for trust and involves balancing empathetic engagement with professional boundaries. However, emotional presence may also challenge these boundaries, creating ethical tensions that require careful attention (Lagerin et al. 2025). This understanding helps patients feel seen, supported and informed, allowing conversations to unfold on their terms (Engel et al. 2023). Trust is fundamental; without it, patients may hesitate to share sensitive thoughts (Höglander et al. 2023). Trust is further strengthened when patients can select whom they confide in, as having their preferences respected contributes directly to their sense of well‐being and safety in sharing sensitive information (Stenman et al. 2024). A theoretical analysis of person‐centred conversation in nursing emphasises that communication must be adapted to each patient's wishes, strengths and capacities (Öhlén and Friberg 2023).

Previous qualitative empirical studies have indicated the importance of confidential conversations (Stenman et al. 2023, 2024, n.d.), yet little is known about the specific circumstances under which they emerge, how they unfold, or why they sometimes fail in everyday practice. Moreover, the interpersonal dynamics that enable or hinder these conversations are insufficiently understood from both the RN's and the patient's perspectives (Höglander et al. 2023; Moran et al. 2024). This study addresses this gap by exploring how interpersonal relationships form the foundation for confidential conversations in palliative care nursing. It examines how RNs create opportunities for confidential conversations and how patients attempt to initiate them, thereby contributing to a deeper understanding of the relational dynamics that support communication at the end of life.

## 3 The Study

### 3.1 Aim

The aim was to explore aspects of interpersonal relationships in palliative care nursing, with a focus on confidential conversations between RN and patients.

### 3.2 Objectives

- To identify and describe how RN create opportunities for confidential conversations with patients.
- To identify and describe how patients attempt to initiate and engage in confidential conversations with RN.

## 4 Methods/Methodology

### 4.1 Design and Theoretical Frameworks

This qualitative study adopts a social constructivist approach, which views reality as socially constructed through human interactions, with knowledge being interpreted in context (Patton 2015). To explore the interpersonal relationship between RNs and patients in palliative care, a focused ethnography was employed. Focused ethnography is a qualitative method involving intensive, targeted observation of specific social situations or activities over a limited time (Knoblauch 2005). It is used across health disciplines to study complex, dynamic interactions, capturing both explicit and implicit aspects (Higginbottom et al. 2013). Social constructivism aligns with focused ethnography, as both emphasise the co‐construction of meaning and knowledge (Knoblauch 2005). Focused ethnography facilitates an in‐depth exploration of how meanings are created and negotiated in natural settings (Higginbottom et al. 2013). By adopting this approach, the study recognises that RN‐patient experiences are co‐constructed through ongoing interactions within palliative care.

Through unstructured observations, field notes and interviews, this study explores the interpersonal dynamics of palliative care, focusing on confidential conversations grounded in person‐centred care, which honours each patient's unique values and preferences (Öhlén and Friberg 2023). These values are further reflected in Oberle and Davies' (1993) theory of supportive care, which emphasises presence, empathetic listening and shared meaning.

The study followed the Consolidated Criteria for Reporting Qualitative Research (COREQ) checklist (Tong et al. 2007; Appendix A).

### 4.2 Study Setting and Sampling

Palliative care in Sweden is provided across hospitals, hospices, nursing homes and in patients' homes, with access based on need rather than prognosis. This enables early referral for symptom management and psychosocial support at various stages of illness (Payne et al. 2022). The study was conducted in specialised palliative care units in the Northern Region of Sweden. This vast and sparsely populated region comprises four counties with a total population of approximately 900,000 inhabitants (Statistics Sweden 2021). Specialised palliative care services are provided in inpatient wards or in patients' homes across both rural and urban areas. The region includes two hospices, one 24‐h care ward and four home healthcare teams.

Purposeful sampling (Patton 2015) was employed to select participants who could provide rich and relevant insights. First, information letters were distributed to managers of nearby units; three managers agreed to participate: two from home care units and one from a hospice care unit. Managers then distributed the study information to RNs who met the inclusion criteria of working in specialised palliative care and were available to take part in observations and interviews (see Table 1 for the demographic profile of RNs). RNs interested in participating registered their interest via email to the first author (T.S.), who then contacted them to provide further study details. Subsequently, RNs identified potential patient participants and informed them about the study. Patients were required to be over 18 years of age, able to understand the study information and to provide informed consent. The average care duration for home care was 36 days, while at the hospice it was 13 days, indicating that patients were in the final stages of life (see Table 2 for the demographic profile of patients).

**TABLE 1:** Demographic profile of Registered Nurses (RN).

*N* = 13	
Gender	
Female/male/other	11/2/0
Age (years)	38–69 (mdn 57)
Year as a RN (years)	5–47 (mdn 20)
Year in palliative care (years)	5–24 (mdn 8)
Working	
Home health care/Hospice	8/5
Education	
Undergraduate RN/specialist RN	2/11

**TABLE 2:** Demographic profile of patients, *N* = 22.

Gender	
Female/Male/Other—prefer not to say	12/10/0
Age (years)	57–92 (median 79)
Performance status EQOG* (0–4)	2–4 (median 3)
Diagnosis	
Cancer/Other	20/2
Current palliative care	
Homecare/Hospice	17/5

* The EQOG describes a patient's level of functioning in daily life from 0 to 5, where 0 is fully functional and five is death (Oken et al. 1982).

### 4.3 Data Collection

The study was conducted from March to October 2024, encompassing 25 observations totalling 28.5 h. Of these, 23 observations were conducted by the first author T.S., while two were performed by a trained research assistant to ensure timely data collection when patients' health deteriorated. Follow‐up interviews were conducted with a total of 16 patients and 24 interviews were conducted with 13 different RNs (see Table 3). Patient non‐participation was attributed to illness, refusal or inaccessibility, while RN dropouts were primarily due to high workload.

**TABLE 3:** Overview of observations and interviews.

Obs	Patient code	RN code	Setting	Others present	Obs duration minutes	Patient interview minutes	RN interview minutes
1	01	01	Home	Partner	50	4	10
2	02	01	Home	None	40	2	4
3	03	02	Home	None	60	4	7
4	04	03	Home	Home care	60	—	11
5	05	03	Home	None	45	5	7
6	06	04	Home	Home care	35	—	13
7	07	04	Home	Partner	45	—	13
8	08	03	Home	None	90	2	8
9	09	03	Home	Partner	75	3	4
10	10	05	Home	Partner	45	2	6
11	11	01	Home	Partner, district nurse	115	2	9
12	12	06	Home	Child and grandchild	60	4	2
13	13	07	Home	District nurse	255	2	12
14	14	08	Home	Partner	70	2	2
15	15	05	Home	Partner, relatives	80	2	7
16	16	05	Home	None	80	4	2
17	17	01	Home	None	85	6	2
18	18	09	Hospice	Assistant nurse	110	—	3
19	19	09	Hospice	Assistant nurse	95	—	4
20	20	10	Hospice	None	50	3	6
21	21	10	Hospice	None	40	4	6
22	20	11 and 12	Hospice	None	35	—	6
23	21	11	Hospice	None	20	—	6
24	22	11	Hospice	Partner, assistants	45	—	6
25	20	13	Hospice	None	20	—	—

Data collection involved unstructured participant observations in patients' homes or hospices, a method well suited for studying complex healthcare interactions. These observations were supplemented by reflective field notes and brief follow‐up interviews with RNs and patients (Fetters and Rubinstein 2019; Knoblauch 2005).

Observations began after consent was confirmed and continued until the end of the visit. Fetters and Rubinstein's ‘3 Cs’ framework Fetters and Rubinstein (2019) guided the observations, allowing for flexible data collection in which the researcher interpreted phenomena based on context and personal insights. The framework, formatted as a protocol (Appendix B), included demographic details and space for field notes. The observer documented the RN and patient interactions. After each observation, brief interviews were conducted with the patient asking, ‘How did you experience today's encounter?’ with probes such as, ‘Can you elaborate?’ RNs were asked the same question, with follow‐up questions focusing on relational reflections. These interviews were included to deepen the understanding of observed interactions and capture participants' perspectives. Only the interviews were digitally recorded as audio files (see Table 3 for an overview of observations and interviews).

Jottings were made during observations to aid recall and were later expanded into detailed field notes (Fetters and Rubinstein 2019). Field notes were rewritten immediately after each observation (Higginbottom et al. 2013). Some participants requested to review the protocol beforehand, while others preferred to read the notes afterwards.

### 4.4 Data Management and Analysis

The research group regularly reviewed the data to confirm that the study objectives were sufficiently addressed and the emerging themes provided rich and relevant insights, ensuring adequate information power. According to Braun and Clark (2021), information power refers to the adequacy of the data in providing meaningful answers to the research questions, emphasising depth and relevance over sample size.

All data were collected and analysed in Swedish to preserve linguistic and cultural nuances. The first author (T.S.) transcribed all interviews verbatim and subsequently translated the data into English, with translations of illustrative quotes cross‐checked within the research team. The data were analysed using reflexive thematic analysis (RTA) as described by Braun and Clark (2021). The research team reviewed the material multiple times to gain a deeper understanding. The data consisted of 138 pages of observation protocols and field notes, as well as 80 pages of interview transcripts. The large volume of data was organised with the aid of the mapping technique described by Braun and Clark (2021). Data were managed and analysed manually using a systematic coding process. Initial codes were generated from the data, focusing on key aspects guided by the study's objectives. These codes were grouped into themes by organising related data, a process that was continuously discussed within the research team. Finally, the analysis was compiled by identifying key themes, illustrating them with examples from the collected data and interpreting the findings in relation to the study's broader context (See Appendix C for examples of this process.).

### 4.5 Ethical Considerations

The Swedish Ethical Review Authority (Dnr 2024‐00341‐02) approved the study in accordance with the Declaration of Helsinki (World Medical Association Declaration of Helsinki 2024). Informed consent was obtained from both patients and RNs. Patients received verbal and written information and provided consent either verbally or in writing, depending on their condition. Cognitive ability was not formally assessed using standardised tools; inclusion was based on the RN's and research team's evaluation of patients' understanding of the study and the consent procedure. Participants were informed that participation was voluntary and that they could withdraw at any point without affecting their care. RNs also provided written consent and were similarly informed of their right to withdraw.

Given the vulnerability of patients with palliative care needs, ethical considerations prioritised dignity, autonomy, well‐being, confidentiality and a respectful care environment. Observations and interviews were designed to minimise disruption to clinical routines. The researcher assumed a non‐participatory role, observing without direct involvement while remaining aware of their potential influence on the setting (Higginbottom et al. 2013).

Sensitivity to patients' emotional and physical states was observed throughout the data collection. Including vulnerable participants was ethically justified by the potential to gain valuable insights into palliative care (World Medical Association Declaration of Helsinki 2024). In cases of participant discomfort, the researcher was prepared to offer immediate support and, if necessary, involve the responsible manager. No participant required such support.

All data were stored in accordance with current data protection regulations on secure, encrypted servers with access restricted to authorised members of the research team. A separate, pseudonymised code key was maintained to safeguard participant confidentiality. Data handling complied with ethical guidelines, GDPR requirements and established best practices in research integrity and security (World Medical Association Declaration of Helsinki 2024).

### 4.6 Rigour and Reflexivity

Rigour was ensured through systematic and transparent data collection and analysis. Multiple data sources—observations, field notes and interviews with patients and RNs—enabled triangulation, thereby strengthening credibility.

Reflexivity was central to maintaining integrity throughout the study. The research team, comprising individuals with diverse experience in palliative care (T.S., B.H., U.N., C.M.J.) and home/psychiatric care (Y.R.), provided rich contextual insights while also bringing potential preconceptions. This diversity supported collective reflection and nuanced theme development. Reflexive practices—including team discussions, documentation and journaling—helped address the influence of prior experience. The researchers' positionality and assumptions in relation to participants' perspectives were actively considered. RTA (Braun and Clark 2021) offered a flexible framework for ongoing reflection on how the researchers' backgrounds shaped the analysis. Awareness of the researchers' presence in the field and its potential impact on data was maintained. Peer feedback and collegial review further enhanced transparency and ensured grounded interpretations.

### 4.7 Findings

**FIGURE 1:**
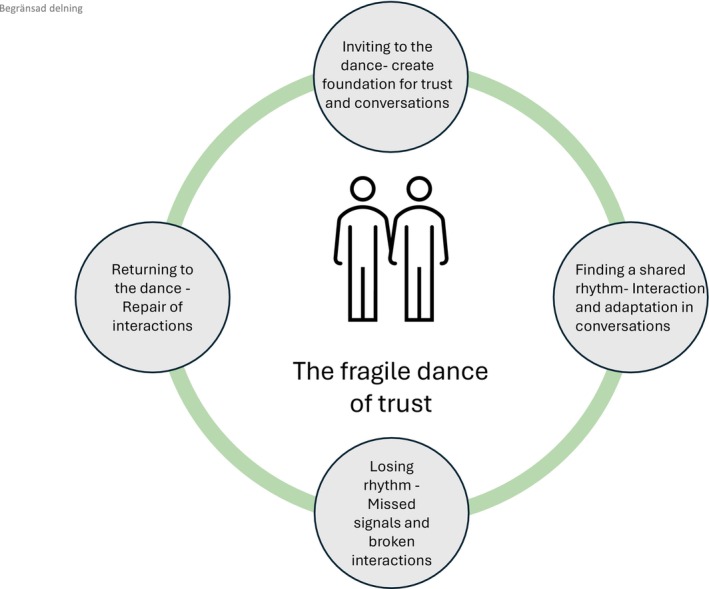
The fragile dance of trust illustrates the main theme of trust in RN–patient conversations in palliative care. The four surrounding circles represent themes describing phases of interaction where trust was built, maintained, lost and restored: Inviting to the dance—create foundation for trust and conversations; Finding a shared rhythm—Interaction and adaptation in conversations; Losing rhythm—Missed signals and broken interactions; Returning to the dance—repair of interactions. These themes should be understood as interconnected components rather than a linear progression; not all themes necessarily occurred in every conversation and they may appear in varying order depending on the interaction. [Colour figure can be viewed at wileyonlinelibrary.com]

The analysis resulted in an overarching theme illustrated through a metaphor: the fragile dance of trust, with four themes further elaborating on different aspects of this fragile nature. The metaphor was constructed during iterative coding and theme generation, as patterns of rhythm, responsiveness and vulnerability became apparent across the data. It captured the shifting, relational nature of interaction between patients and RNs, where trust was continuously negotiated. Each theme was metaphorically represented and is illustrated in Figure 1. The findings, drawn from observations, field notes and interviews, are supported by illustrative excerpts from observations (1–25).

### 4.8 The Fragile Dance of Trust

The foundation of the conversation lay in fragile trust, built, maintained, loosened and restored through words, presence, actions and sensitivity to context. The circumstances interacted, creating space and time for doing, being and unfolding. Observations revealed that factors influencing confidential conversations included the work environment, physical setting, relationship history, family presence and the patient's condition. A sense of safety and trust facilitated conversations, while stress or time pressure often hindered them. Across all interactions, RNs demonstrated attentiveness to verbal and non‐verbal cues, adapting to the patient's pace, body language and emotional state. Silence was an overarching tool used throughout conversations, taking on specific roles in different themes.

In the interviews, patients emphasised that trust was built through consistent trust and confidence, rather than through single actions or conversations. Both patients and RNs identified the RN's competence, knowledge and compassionate presence as key elements in fostering trust. Given the patients' illness and limited time, trust became particularly fragile and RNs expressed concern about losing moments to connect or failing to maintain trust. Both patients and RNs reflected on how essential time and continuity were for sustaining the relationship.

By inviting each other into the dance, conversations found deeper meaning, allowing patients to express their thoughts and feelings. When conversations flowed, trust was strengthened, fostering a shared rhythm. However, obstacles such as interruptions or misunderstandings could disrupt the flow, causing missed signals. Once corrected, the rhythm was restored, creating new opportunities for conversations.

### 4.9 Inviting to the Dance: Create Foundation for Trust and Conversations

Creating foundation for confidential conversations was like inviting someone to dance, with the RN using small talk and presence to initiate dialogue. It was not only about speaking, but also about attentively observing the patient, something RNs described in interviews as a deliberate strategy. Presence and interest were conveyed through actions such as sitting close to the patient, maintaining eye contact and occasionally offering gentle touch. Observations showed that silence in this theme provided space for conversation, signalling the RN's attentiveness and willingness to engage. At times, RNs also incorporated symptom assessment tools or symptom lists, which provided structure while enabling deeper exchanges. In Observation 2, the RN followed up on symptoms, inviting a confidential conversation about fear of deterioration:

RN 01 How are you feeling?

Patient 02 I'm okay, but recently I've been having pain here (points to stomach).

RN 01 Is it constant pain?

Patient 02 No, just at night, toward the morning. And then I think – this is it. The cancer's back, it's over…

RN 01 Do you feel that way when it hurts?

Patient 02 Yes, I give up now. That's when I realise it's the cancer, and it's the end.

RN 01 Could you tell me more about it?

Initiating small talk was essential for connecting the RN and patient beyond illness and symptoms, establishing a foundation for conversation and allowing the RN to pick up on cues that led to deeper exchanges. RNs tailored small talk to make the conversation personal, creating a shared understanding and helping patients feel seen and understood. The balance between words and silence required courage and attentiveness from both parties. Emotional presence fostered a climate, where patients felt that tone and treatment were key to making them feel comfortable talking. Patients also initiated conversation with direct questions, reflections, jokes or hints.

For patients with limited language, for example, aphasia or fatigue, the RN's sensitivity was crucial. Reading small changes in facial expressions and body language became key, rooted in the relationship with the patient, as described by the RNs. Patients valued meeting the same RN and having the option to talk when they wanted. Being remembered created a sense of worth, and the conversations were often seen as: ‘A feeling of becoming friends’ (Patient 01).

### 4.10 Finding a Shared Rhythm: Interaction and Adaptation in Conversation

Once the conversation began, the RN and patient could find a shared rhythm. In their interpersonal relationship, a dynamic interaction emerged, with the conversation shifting between pace, tone and pauses, all shaped in the moment. The RNs described a feeling that something special had occurred: ‘A moment that is difficult to put into words’ (RN 07).

The conversation followed the patient's rhythm and gradually shifted to more personal topics, such as discussions about children and grandchildren, leading to sorrow over leaving them. The RNs adapted their responses. This was seen in Observation 10, where a conversation turned into thoughts about impending death.

RN 05 When you took the sedative earlier today, was it the anxiety in your body you were feeling?

Patient 10 Yes, it's the anxiety in my body.

RN 05 Are there any thoughts causing you concern?

Patient 10 No, it's just how it goes. It feels more like pre‐travel nerves.

RN 05 Are you worried about the journey itself or the destination?

Patient 10 Well… am I forgetting something? But if I have, I'm sure it will be fine.

RN 05 What do you think you might have forgotten?

Patient 10 That's the thing… I don't know. But I'm not worried that it won't be okay.

RN 05 Our role is to make sure the journey is as good as it can be.

Patient 10 Yes. I know. I feel safe.

When the patient expressed frustration or sorrow, the RN responded with a softer or affirming tone. Synchronisation occurred through non‐verbal cues and reading between the lines. When a patient joked or changed the subject, it could indicate sensitivity, requiring careful exploration without overstepping boundaries, a balance RNs found crucial to maintain.

The patient controlled both the pace and the content of the conversation, which could shift or circle back to earlier topics. Slower speech and longer pauses signalled a need for reflection. When sensitive themes such as death or existential concerns emerged, patients required time to process them. In this theme, silence created a space for reflection, enabling patients to explore their thoughts and feelings while the RN adjusted the dialogue accordingly. By remaining attentive and allowing time for reflection, the RN fostered deeper listening and attunement to the patient's needs.

When the RN paused without urgency, stability was created, encouraging the patient to talk. Significant others also influenced the rhythm, supporting the patient or offering reflections. In interviews, patients appreciated when the RN included significant others, contributing to a sense of community and safety. These moments created a shared experience where everyone cooperated.

### 4.11 Losing Rhythm: Missed Signals and Broken Interactions

Despite good intentions, the conversation between the RN and patient could fall out of sync. The imbalance created distance, making it difficult to maintain the flow. Sensitive topics, such as loss or death, could go unnoticed if the conversation ended prematurely due to time constraints, risking the patient's need for continued dialogue unmet. It was noted that the RN sometimes missed cues from the patient who wanted to talk, often due to a focus on their own agenda or practical tasks, as in Observation 17:

RN 01 We visited you the first time 6–7 weeks ago. How do you feel that time has passed?

Patient 17 I have had trouble sleeping, and it's a concern. I want to sleep, at the same time—I don't want to fall asleep because I'm afraid I won't wake up again. I have thoughts that come to me at night, and I lie there thinking. Overall, it doesn't really feel like I have cancer. But it's there in my stomach (places hand on stomach), and it's spread.

RN 01 Do you go out much?

In some situations, if the questions became direct or the conversation progressed too quickly, the patient signalled resistance by changing the subject, responding with irony or irritation, or remaining silent. Periods of silence in this theme could reflect disruption, disengagement, or a protective mechanism when the patient was not prepared to engage. If the RN ended the silence too soon, the conversation risked closing, whereas prolonged silence from the RN could be perceived as withdrawal. External factors such as disruptive noise, colleagues, ringing phones or interruptions from significant others influenced the conversation. This became evident in Observation 7:

The RN clears away the waste after the injection and sits down again, facing the patient.

**RN 04** Do you have any pain?

**Patient 07** No.

**RN 04** No pain. But anxiety?

**Patient 07** Yes.

The husband responds, talking about the patient and her anxiety, mentioning that she rests a little during the day.

**RN 04** [to the patient] Do you sleep through the night?

**Patient 07** Oh, absolutely.

The husband takes over, discussing the nights, home care and expressing dissatisfaction. The RN lets him speak and listens. The patient listens and remains silent.

In a stressful healthcare environment, it was difficult to give full focus, which impacted emotional presence and conversation quality. When the RN was not fully present, the patient's attempts to initiate conversation could be overlooked. RNs experienced frustration and inadequacy, fearing they might say the wrong thing and lose the patient's trust. They aimed to create a safe environment but felt insecure when the conversation shifted or was avoided. Balancing honesty and gentleness were challenging, and they adjusted to the patient. Sometimes, the right words or tools for facilitating confidential conversations were missing, requiring shifts in tone, pacing, or non‐verbal cues depending on the patient's condition.

### 4.12 Returning to the Dance: Repair of Interactions

When the conversation fell out of rhythm, it did not signify that it was lost; rather, it created an opportunity to reassess, adjust and reconnect. When missed signals were corrected, new opportunities for confidential conversations emerged. In interviews, RNs expressed a sense of failure in the conversation, which they then sought to repair. Small but meaningful actions—such as a reassuring glance, an open question, or sitting down to face the patient—could restore the interaction and restart the dialogue.

The RN could step back, reassess, listen more attentively, adjust their approach or shift the topic to repair the interaction. Sometimes, this required letting go of their own agenda to allow the conversation to flow naturally; at other times, it meant adhering to important points and returning to them later. RNs emphasised the importance of reconnecting with patients, reassuring them with statements like, ‘We'll see each other tomorrow’, signalling that the conversation was not over and that there would be further opportunities to talk.

After completing a practical task, the RN could reconnect, helping to restore the rhythm and re‐establish continuity—something patients appreciated, as shared in interviews. A good example of this was observed in Observation 13.

The patient asks questions about the medication pump, the medication inside it and how much has been taken and administered. The RN replaces the cannula and explains what she is doing.

**Patient 13:** Is there enough medication in that to take one's life? Could you use what's in the cassette and take it all at once? It should be possible with the right equipment.

The RN and district nurse discuss that this is not possible. The RN begins by explaining the pump's settings and how they prevent an overdose. The patient clarifies that this is not what he meant and continues trying to make them understand his point. He then quickly changes the subject and starts talking about a TV show. The RN and district nurse finish adjusting the pump and conclude the discussion.

A moment of silence follows.

The RN takes a chair, places it by the bed, sits down facing the patient with both feet on the floor and leans slightly forward.

**RN 07:** I want to revisit the conversation about suicide. Do you have thoughts like that?

Expressing needs and desires was crucial for patients to reinitiate conversation. They often waited for the RN to finish a task before resuming, either directly or through subtle cues such as a change in tone or renewed engagement. Both the RN and the patient could use personal topics to restore flow, with small talk easing tension and making it easier to return to difficult subjects. In this theme silence served as a reparative function, keeping the conversation open and giving the patient space to reflect before continuing.

Minimising disturbances, such as closing a door or lowering the TV volume, improved conditions for conversation. The RN's body language and eye contact signalled engagement and helped restore trust. Significant others also played a role by revisiting topics or fostering a calm atmosphere, creating opportunities to reconnect and find a shared flow.

## 5 Discussion

This study explored interpersonal relationships in palliative nursing, focusing on confidential conversations between patients and RNs. These relationships were grounded in fragile trust, shaped by presence, sensitivity and mutual adaptation. Small talk initiated and sustained connection, while silence invited reflection and signalled shifts in dialogue, reflecting the essence of person‐centred care, where emotional attunement guides interaction (Öhlén and Friberg 2023).

The overarching theme ‘The fragile dance of trust’ and its four interrelated subthemes –‘Invite to the dance,’ ‘Finding a shared rhythm’, ‘Losing rhythm’, and ‘Returning to the dance’ generated through RTA. The metaphor reflected dynamic trust patterns observed during analysis, illustrating how trust is co‐created, disrupted and restored. Our findings demonstrate how trust was negotiated moment to moment under conditions of vulnerability and limited time, supporting a relational ontology of care (Bender 2018) and social constructivist epistemology (Knoblauch 2005).

Trust and interpersonal relationships were reciprocal and essential for confidential conversations. Trust entailed patients' willingness to risk vulnerability (Bell and Duffy 2009), reinforced by the RN's authenticity and presence (Smith et al. 2024). These findings deepen understanding of Oberle and Davies' (1993) theory of supportive care by showing how attuned listening created a safe relational space where patients' emotional experiences could surface. In line with Holopainen et al. (2019), the conversations were not simply about exchanging information, but were transformative and emotionally supportive, demonstrating the importance of relational depth in palliative care. At the same time, trust was fragile. The patient's changing condition, lack of energy and limited time made each meeting more vulnerable. Time became both a reminder of life's finiteness and a practical limitation, making each moment valuable yet fragile (Tarbi et al. 2021). These existential dimensions further highlight the need for relational sensitivity and emotional availability (Oberle and Davies 1993), as well as a person‐centred approach (Öhlén and Friberg 2023).

RNs initiated confidential conversations by engaging in everyday small talk and relationship‐building. Successfully initiating these conversations requires a calm demeanour, privacy and a respectful tone (Hussain 2020; Lagerin et al. 2025). RNs prioritised establishing trust before addressing sensitive topics, with patients generally guiding the pace and depth of the discussion. Contrary to Von Blanckenburg et al. (2022), who advocate for RN‐led initiation, our findings indicated that patients played a more active role than previously assumed. These insights challenged assumptions of asymmetry (Höglander et al. 2023), underscoring the need for relational sensitivity over scripted interventions.

Conversations deepened when RN and patient found a shared rhythm. Non‐verbal communication and presence enabled patients to process experiences at their own pace, also described by Engel et al. (2023). Our findings elaborated on Moran et al. (2021) and Holopainen et al. (2019) by demonstrating that moments of relational alignment fostered emotional closeness and authenticity. In these moments, the RN's courage to listen in silence was not passive, but an act of professional commitment (Stenman et al. 2023). Silence functioned as a relational cue that, when used intuitively, supports reflection and trust (Bassett et al. 2018). This courage, integral to the RN's professionalism, helps foster authentic and meaningful interactions (Holopainen et al. 2019).

However, the rhythm of conversation could be disrupted by missed cues or task‐driven interactions, creating asymmetry. This could disrupt trust and, as a result, patients hesitated or silenced themselves, which has been found to increase anxiety and make re‐engagement more difficult (Wiechula et al. 2016). Consistent with Höglander et al. (2023), this led to communication asymmetry and sometimes avoidance due to emotional strain (Pentecost et al. 2020). Our findings highlighted the emotional labour involved and how its management was integral to sustaining patient‐centred communication.

Our findings showed that RNs often attempted to restore connection. This was facilitated through listening, validation and the gentle reintroduction of sensitive topics, strategies that have also been described by Von Blanckenburg et al. (2022) and Lagerin et al. (2025). Recent research highlights RNs' strong commitment to succeed in these interactions and their persistent desire to repair and sustain the conversation (Stenman et al. 2026). Interestingly, patients in our study also actively tried to re‐engage by referring to earlier discussions, which differed from Von Blanckenburg et al. (2022) concept of ‘I‐Other’ asymmetry, where patients, despite their willingness, expected the RN to take the lead. Patients' concern about burdening staff further underscored the importance of consistently creating relational openings for dialogue.

Intuition was a vital tool for RNs, supporting sensitivity to patients' emotional and physical cues. Small talk emerged as an intuitive, strategic entry point, fostering relational depth and enabling existential dialogue (Macdonald 2016; Tarbi et al. 2021). In our study, small talk was not seen as peripheral but as foundational in building trust and creating space for existential conversations. This supports Cribb et al. (2022), who argue that small talk plays an essential role in relational care, facilitating a shared rhythm and opening the door for deeper, more meaningful dialogues. This embodiment of supportive care includes elements of hope and relational presence (Oberle and Davies 1993).

Silence, though complex, was equally relational. RNs used intuition to discern when silence was a natural part of the conversation, a protective shield or a signal to gently re‐engage. Silence plays a central role in emotional reflection and processing (Wiechula et al. 2016). Misreading silence, especially from the RN, risked conveying detachment (Bassett et al. 2018). This reinforces Moran et al.'s (2024) view of nursing as both technical and relational artistry, where tacit knowledge shapes meaningful care.

### 5.1 Strengths and Limitations of the Study

The study employed a coherent methodological approach, combining unstructured participant observations, field notes and interviews to explore both explicit and implicit aspects of interaction. Data collection in palliative care presented challenges due to patients' frailty, resulting in a time‐consuming process and limited data. Interviews were adapted to patients' conditions, which at times limited conversational depth. To address the risk of rapid deterioration and long travel distances, a trained research assistant conducted two of the 25 observations. This adjustment supported continuity and contributed to triangulation, as the shared observation protocol proved robust and transferable between observers. Consistency was maintained through clear study aims and structured guidelines, which strengthened credibility.

Participant behaviour may have been influenced by the researcher's presence, which could have affected both verbal and non‐verbal responses (McCambridge et al. 2014). To mitigate this risk, prolonged engagement, background presence and reflexive field notes were used. Key informants were informed about the study and some reviewed the protocol beforehand, which may have heightened awareness of relational aspects. However, this was not consistent across participants and no clear signs of altered behaviour were observed. Variations in patients' cognitive status may also have influenced communication and engagement, potentially shaping how interactions were observed and interpreted.

The predominance of women among participating RNs reflects the palliative care workforce in Sweden, supporting transferability within this context. Although differences between the two care settings were not analysed, the use of a shared protocol and consistent focus on interpersonal relationships strengthened the study's trustworthiness. Observations were not audio‐recorded; instead, detailed field notes were taken. This approach facilitated focus on interactions and context, though exact wording may be missing.

Finally, Braun and Clark (2021) RTA enabled both descriptive and interpretative insights. While qualitative analysis is inherently interpretative, reflexivity, triangulation and collegial review strengthened the credibility of findings. It should also be noted that the study was conducted in Sweden and all interactions, field notes and interviews were in Swedish. Applying these findings in other linguistic or cultural contexts requires careful consideration of translation and cultural nuances, as communication styles, expressions of trust and norms around end‐of‐life conversations can differ across settings.

### 5.2 Recommendations for Further Research

Future research could explore how gender dynamics influence confidential conversations and the role of small talk in building trust. Studies are also needed on confidential conversations in high‐tech or time‐pressured environments, including tele palliative care. Organisational research could examine how structural factors and peer support affect nurses' relational care and emotional resilience.

### 5.3 Implications for Policy and Practice

Confidential conversations are a core aspect of high‐quality palliative care. Relational skills, such as attuned listening, silence management and rhythmic sensitivity, should be embedded in education and clinical supervision. Training in communication, cultural competence and ethical sensitivity should also prepare nurses to establish trust and confidentiality in culturally diverse or high‐acuity settings, where relational conditions may vary. Organisational support for protected time, privacy and reflective practice is essential to sustain emotionally demanding communication. Emerging models of tele palliative care require attention to how confidentiality and relational presence can be maintained in digital encounters.

## 6 Conclusion

This focused ethnography provides nuanced insights into the fragile foundation of trust, which is shaped and sustained through subtle adjustments in conversation, presence and actions. The findings show that RN create opportunities for such conversations through small talk, emotional presence and sensitivity to timing and cues. Patients, in turn, often subtly signal readiness and seek connection, relying on RNs to notice and respond. Together, these elements form a delicate process of relational attunement, where trust is continuously tested, affirmed and occasionally repaired.

Healthcare settings may benefit from fostering conditions that support these interactions, such as protected time, relational continuity and environments that enable privacy. It may also be valuable for clinical policies to acknowledge the emotional and ethical significance of confidential conversations and to offer training and support structures that assist RNs in handling them.

Although conducted in a specialist palliative care setting, the findings may resonate across various clinical contexts, including acute wards, home care, long‐term care and primary care, where patients at the end‐of‐life seek emotional connection and trust. These insights have wider clinical relevance wherever compassionate, person‐centred care is prioritised.

## Author Contributions

Tove Stenman: Conceptualization, Data curation, Formal analysis, Investigation, Methodology, Project administration, Validation, Visualisation, Writing – original draft, Writing – review and editing. Bodil Holmberg: Conceptualization, Formal analysis, Methodology, Supervision, Validation, Writing – review and editing. Ylva Rönngren: Conceptualization, Formal analysis, Methodology, Supervision, Validation, Writing – review and editing. Ulla Näppä: Conceptualization, Formal analysis, Methodology, Supervision, Validation, Writing – review and editing. Christina Melin Johansson: Conceptualization, Formal analysis, Methodology, Supervision, Validation, Writing – review and editing.

## Disclosure

Nagoya Protocol Compliance Statement: This study did not involve access to genetic resources or associated traditional knowledge as defined by the Nagoya Protocol. Therefore, compliance with the Protocol is not applicable.

## Ethics Statement

The Swedish Ethical Review Authority, Regional Ethics Committee in Stockholm and Department of Other Research (Dns 2024‐00341‐02) approved the study. All participants received written and verbal study information.

## Consent

They gave informed consent, understanding that participation was voluntary and withdrawal was possible at any time. The transcribed interviews were securely stored on a password‐protected computer, accessible only to the research team, ensuring ethical compliance. Quotes were translated as accurately as possible.

## Conflicts of Interest

The authors declare no conflicts of interest.

## Data Availability

The datasets used and/or analysed during the current study are available from the corresponding author tove.stenman@miun.se under the prerequisite that no sensitive, personal or confidential data is revealed. The data and materials are stored on a password‐protected server at Mid Sweden University.

## Appendix 1 Consolidated Criteria for Reporting Qualitative Studies (COREQ): 32‐Item Checklist

**Table:**

No. item	Guide questions/description	Reported on page no.
Domain 1: Research team and reflexivity		
*Personal Characteristics*		
1. Interviewer/facilitator	Which author/s conducted the interview or focus group?	No. 7
2. Credentials	What were the researcher's credentials? For example, PhD, MD	No. 9
3. Occupation	What was their occupation at the time of the study?	No. 9
4. Gender	Was the researcher male or female?	—
5. Experience and training	What experience or training did the researcher have?	No. 9
*Relationship with participants*		
6. Relationship established	Was a relationship established prior to study commencement?	No. 5–6
7. Participant knowledge of the interviewer	What did the participants know about the researcher? For example, personal goals, reasons for doing the research	No. 5–6
8. Interviewer characteristics	What characteristics were reported about the interviewer/facilitator? For example, Bias, assumptions, reasons and interests in the research topic	No. 9
Domain 2: study design		
*Theoretical framework*		
9. Methodological orientation and Theory	What methodological orientation was stated to underpin the study? For example, grounded theory, discourse analysis, ethnography, phenomenology, content analysis	No. 4–5
*Participant selection*		
10. Sampling	How were participants selected? For example, purposive, convenience, consecutive, snowball	No. 5
11. Method of approach	How were participants approached? For example, face‐to‐face, telephone, mail, email	No. 5–6
12. Sample size	How many participants were in the study?	Tables 1 and 2
13. Non‐participation	How many people refused to participate or dropped out? Reasons?	—
*Setting*		
14. Setting of data collection	Where was the data collected? For example, home, clinic, workplace	No. 6 and Table 3
15. Presence of non‐participants	Was anyone else present besides the participants and researchers?	Table 3
16. Description of sample	What are the important characteristics of the sample? For example, demographic data, date	Tables 1 and 2
*Data collection*		
17. Interview guide	Were questions, prompts, guides provided by the authors? Was it pilot tested?	No. 7 and Appendix B
18. Repeat interviews	Were repeat interviews carried out? If yes, how many?	—
19. Audio/visual recording	Did the research use audio or visual recording to collect the data?	No. 7
20. Field notes	Were field notes made during and/or after the interview or focus group?	No. 6–7
21. Duration	What was the duration of the interviews or focus group?	Table 3
22. Data saturation	Was data saturation discussed?	No. 7
23. Transcripts returned	Where transcripts returned to participants for comment and/or correction?	—
Domain 3: analysis and findings		
*Data analysis*		
24. Number of data coders	How many data coders coded the data?	No. 8
25. Description of the coding tree	Did authors provide a description of the coding tree?	Appendix C
26. Derivation of themes	Were themes identified in advance or derived from the data?	No. 8
27. Software	What software, if applicable, was used to manage the data?	—
28. Participant checking	Did participants provide feedback on the findings?	No. 7
*Reporting*		
29. Quotations presented	Were participant quotations presented to illustrate the themes/findings? Was each quotation identified? For example, participant number	No. 9–17
30. Data and findings consistent	Was there consistency between the data presented and the findings?	No. 9–17
31. Clarity of major themes	Were major themes clearly presented in the findings?	No. 10
32. Clarity of minor themes	Is there a description of diverse cases or discussion of minor themes?	No. 10

## Appendix 2 Observation Protocol – Context, Content, Concepts (3Cs) Adapted From Fetters and Rubinstein (2019)

**Table:**

Date	Start time	End time
Who is present? (code) Registered nurse: Patient:	Who is there (context)?	Comment
Reason for visit (context)		
Describe the room/place (context) Is it cramped? Bright? Dark? A home? A patient room?		
Where is the patient? (context) In bed? Up? Sitting? Lying down? Walking?		
Type of care actions (context)		
The meeting (concept) Do they know each other beforehand? How is the meeting initiated? Who does what?		
Interaction (concept) Eye contact/facial expressions/body language/way of speaking		
Who initiates the conversation? (content)		
Who leads the conversation? (content)		
Content of the conversation (content)		
Emotions (content) Emotional expressions – humour, sadness, harmony		

Fetters and Rubinstein (2019).

## Appendix 3 Examples From the Analysis Process

**Table:**

Data source	Excerpt from data	Codes	Theme
Observation 1	The patient talks about everyday topics such as family, grandchildren, baking, activities and home care. The conversation flows naturally, showing familiarity with the RN. Through small talk and questions about daily life, connection and trust are strengthened. The patient shares her anticipation of being able to go out onto the patio once she feels stronger, which shifts the conversation toward expectations and hope.	Everyday conversation as bridge‐building, mutual flow	Inviting to the dance—create foundation for trust and conversations
RN interview (Observation 12)	The RN emphasises sitting close and patiently waiting for the patient's responses, adapting to their long response latency to create calm and connection.	Adjusting communication to patient's needs; physical positioning (sitting down)	Inviting to the dance—create foundation for trust and conversations
RN interview (observation 13):	The RN focuses on addressing the patient's physical needs first to build trust, enabling the patient to feel safe enough to open emotionally and share thoughts.	Supporting patient; facilitating emotional openness	Inviting to the dance—create foundation for trust and conversations
Patient interview (Observation 11)	The patient feels listened to, appreciates involving family and experiences open, inclusive conversations about everyday life.	Feeling listened to, involving family, open conversations	Inviting to the dance—create foundation for trust and conversations
Observation 22	The patient gives minimal responses, including expressing that ‘it's hard’, but the RN focuses on practical questions, resulting in limited emotional connection and interrupted flow.	Interrupted flow; limited response leading to minimal interaction	Losing rhythm—Missed signals and broken interactions
RN interview (Observation 2)	The RN describes difficulty reading emotional cues due to fluctuating patient moods, causing uncertainty in communication.	Difficulty reading emotional cues; fluctuating patient mood; uncertainty in communication	Losing rhythm—Missed signals and broken interactions
Patient interview (Observation 21)	The patient emphasises that trust is essential; without it, care becomes distressing.	Importance of trust, lack of trust leads to distress	Losing rhythm—Missed signals and broken interactions
